# Breaking biofilm barriers in skin wounds: Membrane-Active antimicrobials in an era of resistance

**DOI:** 10.1016/j.crphar.2025.100249

**Published:** 2025-12-23

**Authors:** Lisa Myrseth Hemmingsen, Nataša Škalko-Basnet

**Affiliations:** a Drug Transport and Delivery Research Group, Department of Pharmacy, University of Tromsø the Arctic University of Norway, Universitetsvegen 57, 9037 Tromsø, Norway; b Natural Products and Medicinal Chemistry Research Group, Department of Pharmacy, University of Tromsø the Arctic University of Norway, Universitetsvegen 57, 9037, Tromsø, Norway

**Keywords:** Bacterial membrane, Biofilms, Biofilm disruption, Chronic wounds, Membrane-active antimicrobials, Pharmaceutical technology

## Abstract

Chronic wounds remain a significant challenge for healthcare systems worldwide, placing a considerable burden on both patients and resources. Their management is further complicated by the persistence of biofilm-forming bacteria and an escalating problem of antimicrobial resistance, both of which restrict the effectiveness of conventional therapies. Antimicrobial compounds with a rapid onset of action and activity that is not solely dependent on bacterial metabolism represent promising alternatives for bacterial and biofilm eradication. Among these, membrane-active antimicrobials (MAAs), including antimicrobial peptides, peptidomimetics, and other membrane-disrupting compounds, constitute a particularly interesting group of agents. Recent investigations have revealed diverse mechanisms through which MAAs compromise biofilm integrity, ranging from permeabilization of bacterial membranes to interference with quorum sensing and extracellular polymeric substances. Furthermore, pharmaceutical innovations such as nanoparticle-based carriers, hydrogel matrices, and scaffold-based delivery systems have shown potential to enhance MAA stability, optimize and prolong release profiles, improve antimicrobial and anti-biofilm efficacy, increase tissue penetration, and mitigate cytotoxicity concerns. By integrating insights from microbiology, materials science, and drug development, this short review aims to outline the challenges posed by biofilms in chronic wounds, appraise the antimicrobial and anti-biofilm activity of MAAs, and discuss how advanced delivery strategies might expand their clinical efficacy.

## Highlights

• Membrane-active antimicrobials offer diverse anti-biofilm mechanisms to tackle antimicrobial resistance.
• Pharmaceutical innovations can overcome their drawbacks and speed progress toward clinical use.
• Deeper insight into their mechanisms and targets is essential for full therapeutic potential.

## Graphical abstract

**Figure:**
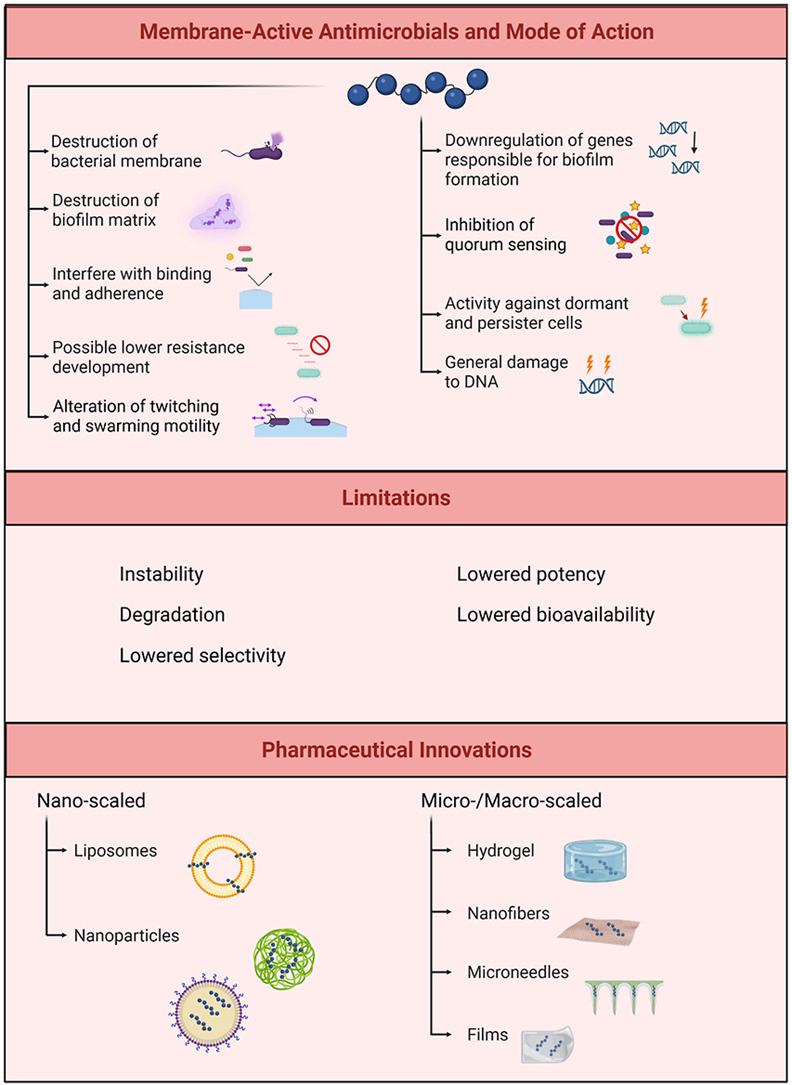

## 1 Introduction

The formation of biofilms is an important factor in the persistence of chronic infections, particularly in non-healing wounds. Biofilms are estimated to occur in approximately 80 % of human infections, substantially increasing the complexity of treatment (Grari et al., 2025). Bacteria embedded within biofilms can display resistance levels up to 1000 times higher than their planktonic counterparts (Goswami et al., 2023). Coupled with the global rise in antimicrobial resistance and the polymicrobial nature of these communities, their eradication remains a considerable therapeutic challenge (Hemmingsen et al., 2021). Among the strategies explored to counteract resistant bacteria and biofilm-associated infections, membrane-active antimicrobials (MAAs) have emerged as a particularly promising class, frequently demonstrating effective anti-biofilm activity (Dias and Rauter, 2019; Hemmingsen et al., 2021).

## 2 Bacterial biofilms in wounds

**Fig. 1:**
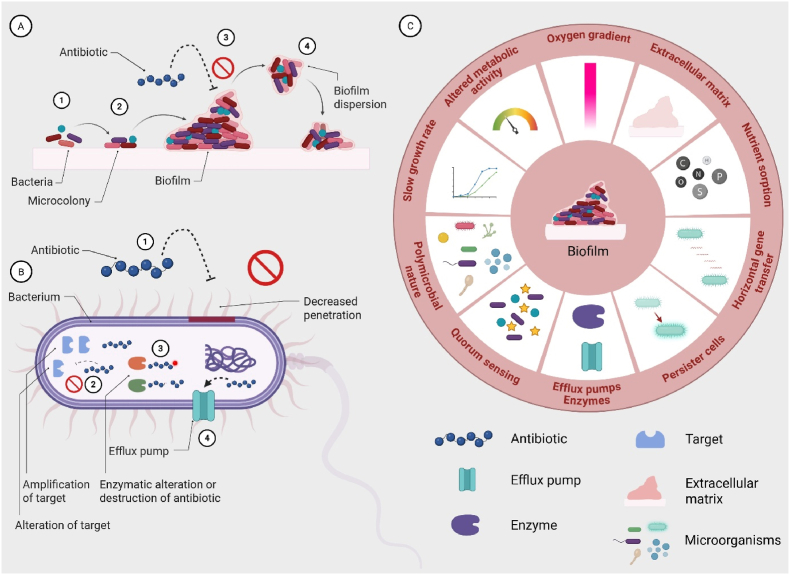
A) Four stages of biofilm formation: 1) attachment 2) formation of microcolonies, 3) biofilm maturation, 4) detachment/dispersion. B) General resistance mechanisms: 1) decreased penetration of antibiotics, 2) alteration or amplification of target, 3) enzymatic alteration or destruction of antibiotic, 4) increase in efflux pumps. C) Common factors found in biofilms leading to resistance or increased bacterial survival in the biofilm community. Adapted from “Polymicrobial Biofilm 2”, by BioRender.com (2025). Retrieved from https://app.biorender.com/biorender-templates.

*Staphylococcus aureus* and *Pseudomonas aeruginosa* are, among others, recognized as major contributors to biofilm formation (Goswami et al., 2023). Biofilm development is commonly described in stages: attachment (reversible and irreversible), microcolony formation, maturation, and detachment or dispersion (Grari et al., 2025), as schematically illustrated in Fig. 1A. In the initial stage, bacteria reach the surface either passively, as in the case of *S. aureus*, or actively via pili or flagella, as observed for *P. aeruginosa* (Geiger et al., 2024; Joo and Otto, 2012). Upon contact, bacteria adhere to host proteins through specific surface-binding adhesins. This attachment activates bacterial signalling pathways that initiate biofilm formation, culminating in the production and maturation of the extracellular polymeric substance (EPS) matrix. In the final stage, bacteria detach from the mature biofilm (Grari et al., 2025). The EPS matrix provides structural integrity and mechanical support to embedded bacteria, while also serving as an additional protective barrier that complements classical antimicrobial resistance mechanisms, as illustrated in Fig. 1B.

Chronic wound biofilms are often polymicrobial, with species interacting synergistically or competitively within a shared matrix. These interactions enhance resilience, increase EPS heterogeneity, and elevate antimicrobial tolerance via shared resistance mechanisms and metabolic cooperation. Effective therapies should therefore target multiple species, penetrate complex EPS matrices, and address metabolically diverse populations (Jo et al., 2022).

**Fig. 2:**
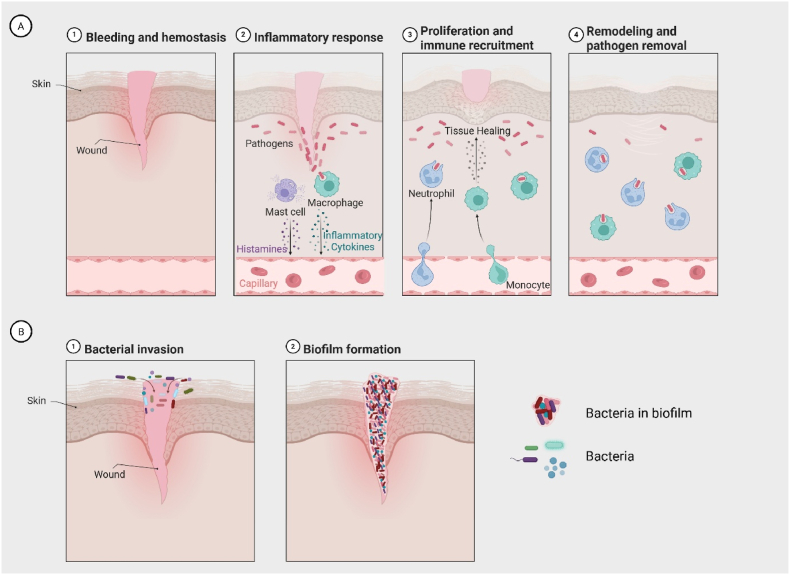
A) Phases of wound repair and associated immune processes: 1) Bleeding and haemostasis: vascular injury triggers platelet aggregation and clot formation to limit blood loss, 2) Inflammatory response: mast cells release histamine, macrophages secrete inflammatory cytokines, and circulating leukocytes are recruited to the wound, 3) Proliferation: fibroblasts, keratinocytes, endothelial cells, and macrophages promote granulation tissue formation, angiogenesis, and re-epithelialization, 4) Remodelling and resolution: macrophages and other immune cells support extracellular matrix remodelling, remove remaining debris, and orchestrate the resolution of inflammation. Note: although depicted as a linear sequence, the wound healing cascade and its associated inflammatory responses are highly dynamic, with stages that frequently overlap and influence one another. B) Bacterial contamination and biofilm development in wounds: 1) Bacterial invasion: planktonic bacteria penetrate the damaged tissue and colonize exposed surfaces, 2) Biofilm formation: microorganisms aggregate, embed in an extracellular matrix, and establish a mature biofilm, protecting them from host defences and antimicrobial agents, thereby complicating eradication and delaying wound healing. Adapted from “The Inflammatory Response”, by BioRender.com (2025). Retrieved from https://app.biorender.com/biorender-templates.

Biofilm-associated resistance arises through multiple mechanisms (Fig. 1C). The matrix acts as a physical barrier, while nutrient and oxygen gradients create heterogeneity, generating persistent or dormant cells with altered metabolism (Cotten Katherine and Davis Kimberly, 2023). Elevated horizontal gene transfer promotes resistance via efflux pumps and enzymatic modification, and enhanced nutrient sorption facilitates resource retention. Quorum sensing further coordinates biofilm behaviour (Grari et al., 2025; Musiol, 2023). Collectively, these factors sustain biofilm resilience, prolong the inflammatory phase, and delay progression through haemostasis, proliferation, and maturation (Fig. 2A and B) in the wound healing cascade (Goswami et al., 2023).

## 3 Membrane-active antimicrobials

**Fig. 3:**
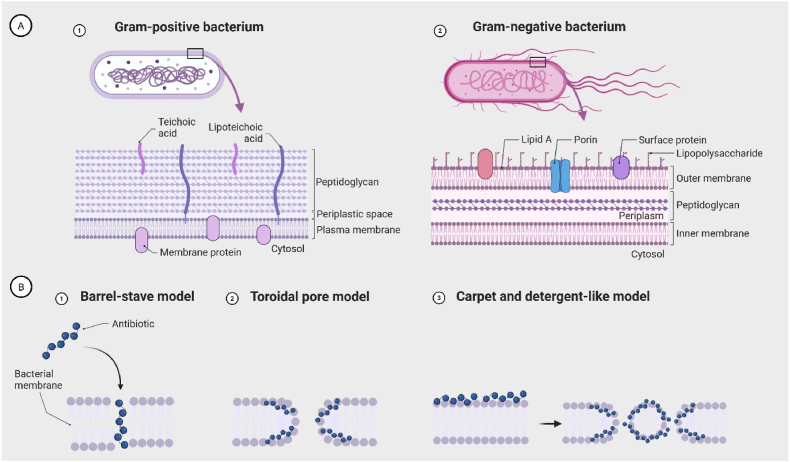
A) Schematic representation of bacterial cell envelope structures. 1) Gram-positive bacteria possess a thick peptidoglycan layer containing teichoic and lipoteichoic acids, a single plasma membrane, and membrane proteins embedded in the cytoplasmic membrane. 2) Gram-negative bacteria feature a more complex envelope with an outer membrane containing lipopolysaccharides, surface proteins, porins, and Lipid A, a thin peptidoglycan layer within the periplasmic space, and an inner plasma membrane. B) Common mechanisms of antibacterial activity of membrane-active antimicrobials (MAAs): 1) Barrel-stave model: MAAs insert into the membrane and form transmembrane channels resembling a barrel. (2) Toroidal pore model: MAAs induce a continuous bending of the lipid bilayer, forming pores lined by both the MAA and lipid head groups. (3) Carpet and detergent-like model: MAAs cover the membrane surface like a “carpet” and disrupt membrane integrity, ultimately leading to formation of micelles or lysis of the lipid bilayer. Adapted from “Bacteria cell wall structures: Gram positive vs Gram negative”, by BioRender.com (2025). Retrieved from https://app.biorender.com/biorender-templates.

To counteract bacterial resistance, antimicrobial agents that act independently of, or are not solely reliant on, bacterial metabolic activity offer a promising therapeutic approach. Membrane-active antimicrobials (MAAs), smaller molecules that mimic host defence or antimicrobial peptides (AMPs), offer compelling alternatives to conventional compounds. The MAAs often exhibit broad-spectrum, rapid activity, and low risk of resistance due to their non-specific mechanisms of action (Zhou et al., 2020). Their cationic charge facilitates binding to teichoic acids in Gram-positive and lipopolysaccharides (LPS) in Gram-negative bacteria (Fig. 3A), destabilizing membranes and inducing bacterial death (Gera et al., 2022).

Several mechanisms have been proposed for AMP and MAA activity. The primary antibacterial modes, illustrated in Fig. 3B, include the barrel-stave, toroidal pore, and carpet and detergent-like models (Zhou et al., 2020). In each, cationic regions mediate electrostatic membrane binding, while hydrophobic domains facilitate penetration and disruption.

## 4 Membrane-active antimicrobials and biofilms

The MAAs include a broad range of compounds of natural or synthetic origin, from plant-derived polyphenols and conventional antiseptics like chlorhexidine to small AMPs and peptidomimetics. These compounds originate from diverse sources, showing a wide variety of chemical structures and biological activities that could target microbial membranes. Naturally occurring MAAs are often produced by plants, bacteria, fungi, and marine organisms (Li et al., 2021a; Macedo et al., 2021; Mohan et al., 2023), providing a rich selection of compounds that have evolved to disrupt microbial membranes as a defence mechanism. Plant-derived MAAs include polyphenols, flavonoids, and essential oils (Panda and Duarte-Sierra, 2022), many of which have been shown to interact with and destabilize bacterial lipid bilayers (Hashempur et al., 2025). Synthetic MAAs comprise chemically designed small molecules and conventional antiseptics, which are optimized for stability, potency, and activity against biofilms (Zhou et al., 2020). In addition, engineered MAAs involve rationally modified peptides and peptidomimetics, which are tailored to improve membrane selectivity, reduce toxicity, and enhance resistance to enzymatic degradation (Li et al., 2021b).

This diversity in origin not only highlights the availability of MAAs from multiple sources but also shows their versatility in application, ranging from topical antiseptics to systemic antimicrobial strategies. A collective feature of these compounds is their ability to interact with bacterial membranes, leading to destabilization, increased permeability, and disruption of biofilms (Ganesan et al., 2023). By targeting the membrane, which is essential for microbial viability, MAAs offer a mechanistically distinct and often complementary approach to conventional antimicrobial therapies (Oliveira Júnior et al., 2025).

Among natural MAAs, quercetin has shown anti-biofilm and anti-virulence effects against *P. aeruginosa* and methicillin-resistant *S. aureus* (MRSA) in a study by Vijayakumar et al. In a dose-dependent manner, quercetin significantly reduced biofilm biomass, disrupted the structural integrity of the biofilm, and suppressed EPS synthesis and protease activity. Quercetin also impaired *P. aeruginosa* swarming and downregulated quorum sensing genes as well as MRSA virulence and biofilm genes, thereby limiting adhesion, toxin production, and biofilm stability (Vijayakumar et al., 2025). Moreover, quercetin might alleviate inflammation, such as that induced by LPS (Aggarwal et al., 2025). Similarly, cannabinoids, particularly cannabigerol, have demonstrated anti-biofilm activity against MRSA, inhibiting biofilm formation, eradicating mature biofilms, permeabilizing membranes, and eliminating persister cells (Farha et al., 2020).

Chemical modification of molecular scaffolds could yield compounds with potent anti-biofilm activity. Suresh et al. developed dihydropyrrol-2-one derivatives that minimally affected *P. aeruginosa* viability but strongly inhibited quorum sensing, representing promising compounds that disarm pathogens rather than kill them. Compounds 10g and 9e suppressed LasR signalling by over 70 % and reduced pyocyanin production, indicating downregulation of virulence pathways. As LasR regulates motility and biofilm formation, these results suggest potential interference with biofilm development, though this was not directly assessed (Suresh et al., 2025).

Small AMPs and AMP mimics often exhibit potent activity against bacterial membranes and biofilms. In *Acinetobacter baumannii*, the peptide KHS-Cnd inhibited biofilm formation at sub-minimum inhibitory concentrations (sub-MIC) and partially disrupted mature biofilms. It reduced motility in a strain-dependent manner, strongly impairing surface movement, abolishing twitching in some strains, and transiently reducing it in others, suggesting interference with early biofilm establishment by limiting adhesion and surface movement, thereby reducing colonization, biofilm maturation, and virulence (Artini et al., 2025). Similarly, in *P. aeruginosa*, AMP 1037 inhibited biofilm formation at sub-MIC levels while increasing bacterial death, modulated motility-related gene expression by downregulating swarming and upregulating twitching, and suppressed other biofilm-associated genes, potentially promoting detachment and limiting surface colonization (de la Fuente-Núñez et al., 2012).

Wang et al. synthesized scorpion-like peptidomimetics that inhibited biofilm formation in MRSA and *Escherichia coli* in a concentration-dependent manner, with membrane disruption confirmed by fluorescence and transmission electron microscopy. These compounds exhibited minimal resistance development, as MIC values remained stable over 14 passages, unlike ciprofloxacin (Wang et al., 2021). Furthermore, Zhang et al. reported that the AP138-derived peptide A24 potently inhibited multidrug-resistant *S. aureus*, reducing early biofilm formation by up to 92 % and mature biofilms by 53–60 %, while killing persister cells at rates up to 99.9 %. Mechanistic studies showed that A24 disrupts bacterial membranes, increases permeability and depolarization, causes potassium leakage, and induces metabolic perturbations, including ATP accumulation and reactive oxygen species (ROS) generation, promoting dormant bacteria transition prior to rapid death. Serial exposure over 30 days led to only a fourfold MIC increase, highlighting low resistance potential. The AMP also demonstrated high physiological stability and rapid bactericidal activity, outperforming AP138 and conventional antibiotics (Zhang et al., 2024). Shi et al. developed the synthetic peptide LI14, which prevented biofilm formation, eradicated established biofilms, and killed persisters. Furthermore, LI14 binds bacterial membranes, including LPS (Fig. 3) and phospholipids, causing pore formation, cytoplasmic leakage, reduced membrane fluidity, disruption of the energy supply, impaired motility, and altered metabolism, with transcriptomic analysis confirming modulation of energy metabolism and membrane integrity genes (Shi et al., 2022).

**Fig. 4:**
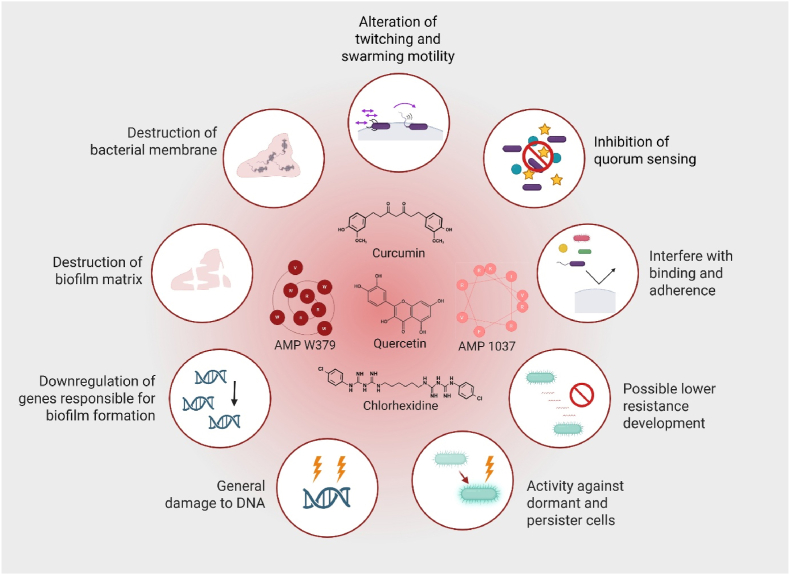
Mechanisms of membrane-active antimicrobials (MAAs) in biofilm-embedded bacteria and bacterial biofilms. The MAAs exert multiple actions that could disrupt biofilm formation and maintenance. These include direct destruction of bacterial membranes, interference with bacterial adhesion and binding, and disruption of the extracellular biofilm matrix. Furthermore, the MAAs could alter motility behaviours such as twitching and swarming, inhibit quorum sensing, and downregulate genes responsible for biofilm development. Additional mechanisms include general DNA damage, activity against dormant and persister cells, and a reduced potential for resistance development. Representative compounds such as curcumin, quercetin, AMP 1037, AMP W379, and chlorhexidine are depicted as examples of compounds potentially targeting these processes. The figure summarizes the multifaceted ways in which MAAs compromise bacterial survival and biofilm integrity. Created in BioRender.com.

The anti-biofilm mechanisms of MAAs are diverse and complex. While the key pathways are illustrated in Fig. 4, additional mechanisms have also been reported in the literature.

## 5 Pharmaceutical innovations

The clinical translation of MAAs, particularly AMPs and AMP mimics, remains limited. Many face challenges such as instability, degradation, low selectivity, limited efficacy, and poor bioavailability, which hinder their clinical advancement (Gera et al., 2022; Wu et al., 2024). These issues can be addressed through drug delivery systems and other pharmaceutical innovations, which could enhance MAA application, retention, and controlled release, thereby improving therapeutic efficacy (Hemmingsen et al., 2021; Meng et al., 2025; Neff et al., 2020).

Here, we highlight some of the promising pharmaceutical innovations designed to improve the stability, efficacy, and delivery of MAAs, offering new strategies to enhance their potential.

Meng et al. evaluated a drug delivery system combining curcumin with a zeolitic imidazolate framework (ZIF-8@CCM) against *S. aureus*. The ZIF-8@CCM exhibited *in vitro* antibacterial activity, including killing intracellular bacteria, and significantly reduced biofilm formation. Scanning electron microscopy imaging and malondialdehyde assays indicated membrane disruption, causing folding, deformation, and lipid peroxidation, whereas curcumin or ZIF-8 alone had minimal effects. The system enhanced curcumin release profile and local activity under acidic conditions, mimicking the infection microenvironment, and demonstrated concentration- and time-dependent bactericidal effects (Meng et al., 2025).

Chlorhexidine-liposomes embedded in a chitosan hydrogel were developed to target *S. aureus* and *P. aeruginosa* biofilms. The system prolonged chlorhexidine release to prevent bacterial regrowth *in vitro*, optimized pH conditions relevant to the wound environment, and potentially enhanced bacterial interactions. The combination of chitosan and chlorhexidine improved biofilm inhibition and eradication of biofilms in both species (Hemmingsen et al., 2021).

Various strategies for delivering AMPs and AMP mimics have been explored against biofilm-producing bacteria. Neff et al. employed a chitosan hydrogel to deliver the small AMPs ASP-1 and ASP-2 against MRSA, *P. aeruginosa*, and *A. baumannii* in *ex vivo* biofilm models on porcine skin. The hydrogel-embedded AMPs achieved complete or near-complete biofilm eradication for all three strains and provided prolonged AMP release over several days, extending bacterial exposure and enhancing efficacy (Neff et al., 2020).

Rashki et al. developed chitosan nanoparticles loaded with LL37 (CS/LL37-NPs) to enhance *in vitro* antibacterial and anti-biofilm activity against MRSA. The nanoparticles showed high encapsulation efficiency and sustained LL37 release, prolonging antibacterial effects compared to the free AMP. The CS/LL37-NPs inhibited biofilm formation by around 68 % and reduced mature biofilm viability. Mechanistically, the nanoparticles interacted with negatively charged bacterial membranes, causing disruption and morphological changes, while sustained release improved biofilm matrix penetration. Gene expression analysis revealed downregulation of icaA, a key regulator of polysaccharide intercellular adhesin synthesis, indicating interference with biofilm development. Encapsulation of LL37 in chitosan nanoparticles thus enhanced membrane binding, prolonged antimicrobial activity, disrupted bacterial membranes, inhibited motility-dependent biofilm formation, and modulated biofilm-related gene expression (Rashki et al., 2022).

Microneedle-based delivery systems show promise in overcoming biofilm-associated barriers. Su et al. developed near-infrared light-responsive microneedle patches that released the AMP W379 on demand to treat MRSA wound biofilms. The system achieved nearly complete AMP release after repeated irradiation and eradicated biofilms *ex vivo* on human skin tissue and *in vivo*, outperforming free AMP or uncoated patches. The AMP activity combined with local hyperthermia disrupted bacterial membranes and biofilm structure (Su et al., 2023).

**Fig. 5:**
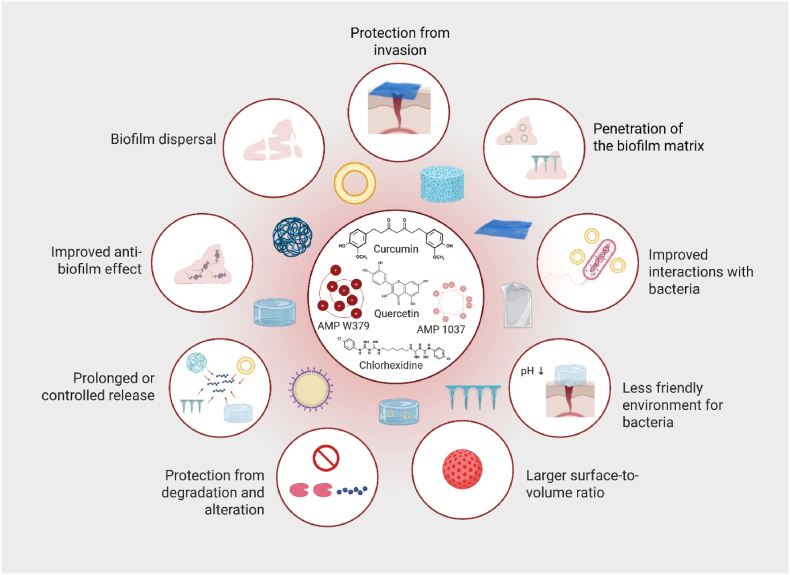
Pharmaceutical innovations to improve membrane-active antimicrobial (MAA) therapy. Schematic representation of potential drug delivery systems and scaffolds for MAAs, highlighting their multiple advantages in combating biofilm-associated infections. The figure illustrates important strategies, including protection from degradation and alteration, prolonged or controlled release, larger surface-to-volume ratio, and improved interactions with bacteria. Additional mechanisms shown are enhanced penetration of the biofilm matrix, biofilm dispersal, protection from invasion, creation of a less favourable environment for bacterial growth (e.g., pH modulation), and overall improvement of anti-biofilm effects. Representative MAAs depicted in the centre include curcumin, quercetin, AMP W379, AMP 1037, and chlorhexidine. The figure was created in BioRender.com.

Moreover, Wu et al. developed a DNA nanotube (DNT) delivery system for the AMP RP557, forming the RP557@DNT nanocomplex. This system enhanced bactericidal activity against multidrug-resistant *S. aureus* and *P. aeruginosa in vitro* and *in vivo*. The RP557@DNT bound bacterial membranes, induced morphological damage, triggered cytoplasmic leakage, and caused DNA damage, achieving near-complete bacterial killing compared to free RP557. In a murine subcutaneous abscess model, it reduced bacterial loads, promoted tissue repair, and resolved inflammation, with favourable biosafety and biodegradability. While biofilm formation was not directly tested, these mechanisms suggest potential efficacy against biofilm-associated infections (Wu et al., 2024). Potential advantages of drug delivery systems and scaffolds for treating biofilm-associated wounds with MAAs are summarized in Fig. 5.

The use of MAAs offers a promising strategy against resistant bacteria and bacterial biofilms, particularly in chronic wounds. Their clinical translation might be limited by instability and low bioavailability. Integrating MAAs with pharmaceutical innovations could enhance stability, increase bioavailability, and facilitate progression into the clinical pipeline. Further elucidation of their mechanisms and identification of specific targets are needed to develop tailored delivery systems for targeted interventions.

## CRediT authorship contribution statement

**Lisa Myrseth Hemmingsen**: Conceptualization, investigation, writing – original draft, writing – review and editing, visualization. **Nataša Škalko-Basnet**: Conceptualization, investigation, writing – original draft, writing – review and editing, funding acquisition.

## Declaration of competing interest

The authors declare that they have no known competing financial interests or personal relationships that could have appeared to influence the work reported in this paper.

## Data Availability

Not applicable.
